# Editorial for Special Issue on “Regulation and Effect of Taurine on Metabolism”

**DOI:** 10.3390/metabo12090795

**Published:** 2022-08-26

**Authors:** Teruo Miyazaki, Takashi Ito, Alessia Baseggio Conrado, Shigeru Murakami

**Affiliations:** 1 Joint Research Center, Tokyo Medical University Ibaraki Medical Center, Ami 300-0395, Ibaraki, Japan; 2 Faculty of Biotechnology, Fukui Prefectural University, Eiheiji 910-1195, Fukui, Japan; 3 National Heart and Lung Institute, Imperial College London, Norfolk Place, London W2 1PG, UK

Taurine (2-aminoethanesulfonic acid) is well known to be abundantly contained in almost all the tissues and cells of various mammals, fish, and shellfish [1]. This abundance of taurine is maintained in two major ways. One is endogenous biosynthesis from sulfur-containing amino acids, and the methionine-cysteine metabolic pathway, which mainly occurs in the liver and some other organs including the kidneys, brain, and adipose tissue [2,3]. The second is an exogeneous mechanism involving a specific transporter, the taurine transporter (TAUT/SLC6a6), which facilitates the intestinal absorption of taurine from dietary animal protein and cellular uptake from circulating blood [4]. Taurine has been reported to play wide-ranging physiological and pharmacological roles, from being involved in basic cell processes such as differentiation, growth, and aging [5,6] to being implicated in the prevention and therapy of liver and heart diseases and inherited metabolic disorders, as well as the benefits of exercise in healthy individuals [6]. In the Special Issue entitled *Regulation and Effect of Taurine on Metabolism* published in the *Metabolites* journal, eight original and two review articles introduce the newest findings of taurine on certain metabolism in humans and animals such as fish.

Taurine is well-known to be an essential nutrient for fetuses and infants during the development and growth of cells and tissues. The ability to biosynthesize taurine is low in the first period of life, and therefore, external intake is essential. Tochitani reviewed the importance of taurine transfer via the placenta and breast milk from the mothers to the fetuses and infants of mammalians, focusing on regulatory mechanisms [7]. During pregnancy, maternal blood circulating in the placenta is concentrated with taurine, allowing its efficient transfer to the fetus’ blood through TAUT present in the placenta. After delivery, taurine is provided to the neonate through breast milk, and its content is the highest during the first days of lactation. In the rat mammary gland, the expression of TAUT and key biosynthetic enzymes is increased during pregnancy and early lactation. The taurine transferred into the fetus and infant are mostly delivered to the brain across the blood–brain barrier (BBB) by TAUT, where it behaves as an inhibitory neurotransmitter—an agonist for γ-aminobutyric acid (GABA) and glycine receptors. Consequently, it promotes the structural and functional maturation of the brain during these periods. In this review, Tochitani also discussed the importance of taurine as a determining factor for health and disease later in life [7].

In addition to its role in the fetal and infant periods, taurine is an important factor for functions in the brain as well as other organs, such as the skeletal muscles, of adults. Watanabe et al. evaluated the influence of taurine depletion on anxiety-like behavior, skeletal muscle function, and body growth using *Taut*-KO mice [8]. In an elevated plus maze test, *Taut*-KO mice were observed to have decreased anxiety-like behavior and difficulties in making decisions underlying an approach-avoidance conflict and risk assessment. The hearing ability was also reduced in *Taut*-KO mice. In addition, a reduction in muscular endurance and lower body growth were observed during the development period from postnatal day 0 until day 60. This suggested that taurine depletion could lead to imbalances in muscular energy production and induce the anorexigenic effect of an adipose-cell-released hormone, leptin, which mediates satiety through the STAT3 pathway. This was confirmed by the phosphorylation of STAT3 being very strongly induced in the brains of the *Taut*-KO mice. Furthermore, Watanabe et al. suggested a possibility that the lower skeletal muscle functions in *Taut*-KO mice could be affected by the activation of STAT3 [8].

Elhussiny et al. evaluated the role of taurine in the brain in the regulation of body temperature and stress behavior in a neonatal chick social isolation stress model induced by the intracerebroventricular (ICV) injection of corticotropin-releasing factor [9]. This factor modulates stress-related effects in the central nervous system [10] and is an indicator of hypothalamic–pituitary–adrenal axis activity [11]. In the neonatal chick model, the ICV injection of taurine alleviated the rectal hyperthermia and stress behaviors such as the number of distress vocalizations and durations of active wakefulness/sleeping postures. In the brains, taurine administration also decreased glutamate, cystathionine, and cysteine, which are substrates and precursors of the antioxidant molecule glutathione. Moreover, it decreased leucine, isoleucine, and alanine, which are related to glutamate synthesis, suggesting that taurine enhanced glutathione synthesis, regulating the stress response through inducing sedative and hypnotic effects.

Similar to the transport of taurine across the BBB by TAUT, taurine was observed to be transported across the blood–testis barrier (BTB) through TAUT, which tightly limits the transport of molecules from the circulating blood into the testis. Taurine is abundant in human semen (where it is 10-fold higher than in the blood) and is suggested to protect the motility and form of sperm from oxidative stress in the testis, where antioxidant enzymes are weakly expressed [12]. Kubo et al. investigated the blood-to-testis transport of taurine using an integration plot analysis in mice and a mouse-derived Sertoli cell line (TM4 cell) [13]. In this analysis, to evaluate the apparent influx clearance, [^3^H]taurine was injected into the mouse’s internal jugular vein and transported into the testis from the circulating blood (approximately 3-fold more than that of a non-permeable paracellular transport marker: [^14^C]D-mannitol). In TM4 cells, the uptake of [^3^H]taurine was time- and dose-dependent, and also Na^+^- and Cl^−^-dependent, but was inhibited by other substrates of TAUT such as β-alanine. This study also confirmed the presence of the *Taut* gene and its protein expressions in the mouse testis and TM4 cells as well as the distribution of its protein in the seminiferous tubules of the mouse testis. The findings of Kubo et al. [13] showed that the higher abundance of taurine in semen is due to transport across the BTB by TAUT. 

Taurine is reported to prevent and improve metabolic diseases as obesity, diabetes, and fatty liver in animal models and humans [14,15]. In this Special Issue, Murakami et al. [16] and Chen et al. [17] describe the ameliorative effects of oral taurine administration on diabetes and fatty liver in a model mouse, and high-fat-diet-induced fatty liver in fish, respectively. Satsu et al. [18] analyzed the mechanism underlying the enhancing effect of taurine on the transcriptional activity of thioredoxin-interacting protein (TXNIP), whose regulatory effects protect against metabolic diseases including diabetes and hypertension and inflammatory diseases including ulcerative colitis [19,20].

In addition to the evidence for beneficial effects of taurine administration on type 2 diabetes animal models, Murakami et al. evaluated the effect of taurine on streptozotocin (STZ)-induced type 1 diabetic mice by focusing on glucose metabolism and oxidative stress [16]. Chronic oral taurine administration alleviated STZ-induced hyperglycemia and hyperketonemia and maintained hepatic glycogen content, with the upregulation of the hepatic glucose transporter (*Glut2*) gene and suppression of oxidative stress in the liver. 

Chen et al. [17] investigated the mechanisms underlying the attenuative effects of dietary taurine administration on fatty liver in groupers (*Epinephelus coioides*). Taurine supplementation significantly inhibited the increases in lipid content and tissue-weight-to-body-weight ratio in the liver induced by a chronic high-fat diet. According to transcriptomic analysis, 160 (72 up- and 88 downregulated) differentially expressed genes (DEGs) were induced by the high-fat diet, and 49 (26 up- and 23 downregulated) of them were identified as being affected by taurine supplementation. Among them, the enriched DEGs involved the upregulation of the bile secretion pathway, fatty acid β-oxidation, and taurine synthesis, and the downregulation of phospholipase D signaling and glycolysis were suggested to be possible mechanisms of the attenuative effect of dietary taurine on high-fat-diet-induced fatty liver in fish. This evidence supports the idea that taurine could improve the efficiency of the aquaculture of fish using high-fat feeds by protecting them against fatty liver syndrome.

Through reporter assays, Satsu et al. confirmed that taurine increased Est-1 protein’s binding to the TXNIP promoter region by the identification of the taurine response element region using human intestinal Caco-2 cells transfected with mutation vectors. Furthermore, taurine was also confirmed to increase the phosphorylation at Thr38 of the Ets-1 protein and activate the ERK1/2 pathway by enhancing phosphorylation; the upregulation of TXNIP gene expression was not observed with p38 or JNK, which also belong to the MAP kinase family. Finally, Satsu et al. suggested that unknown taurine receptors that recognize intracellular/extracellular taurine, leading to ERK–Est-1 activation, might exist in the intestinal cells [18]. On the other hand, the GABA and glycine receptors in the brain are well-known to be taurine receptors [21].

Merck and De Paepe reviewed the role of taurine in skeletal muscle function and its potential support in the treatment of Duchenne muscular dystrophy (DMD) [22]. The authors reviewed what is known about taurine’s role in the physiological functions of skeletal muscles, including the impact of taurine depletion in skeletal muscles in *Taut*-KO mice and in mice treated with the TAUT antagonist guanodinoethane sulfonate. Furthermore, they discussed the roles of taurine in osmotic homeostasis, protein and membrane stabilization, oxidative stress, mitochondrial protein synthesis, and calcium homeostasis in skeletal muscle cells. Subsequently, the pathological characteristics of taurine in an X-chromosome-linked muscular dystrophy (mdx) mouse model and DMD patients were introduced, and current findings on the effects of taurine supplementation on the muscle force, oxidative stress, inflammation, E–C coupling, and histopathological characteristics in the mdx mouse model were discussed. In addition, the synergistic effects of taurine treatment with a corticosteroid (α-methylprednisolone) on muscle strength and muscle atrophy in the mdx mouse were reviewed, including the counteractive effects of taurine on the side effects of a corticosteroid (dexamethasone), such as muscle atrophy and mitochondrial dysfunction in bone.

Although taurine is known not to be metabolized as an end-product of the methionine–cysteine metabolic pathway, the amino group of taurine conjugates with a short-chain fatty acid acetate that is a product of alcoholic detoxification in the liver and, consequently, is converted to *N*-acetyltaurine (NAT) [23]. Miyazaki et al. showed, in a human study, that taurine prevents the accumulation of mitochondrial acetyl-CoA metabolized from acetate derived from the liver in the skeletal muscles during endurance exercise, through the production of the taurine derivative NAT, which conjugates with the excess acetate and promotes its excretion from the skeletal muscle into the urine [24].

Another type of taurine derivative, *N*-chlorotaurine (also known as taurine chloramine; TauCl), is formed by the reaction of the amino group of taurine with excess hypochlorous acid produced via a halide-dependent myeloperoxidase system in phagocytic cells, especially neutrophils and macrophages, where the intercellular taurine concentrations reach 50–100 mM (600-fold higher than those in the plasma). TauCl acts as an anti-inflammatory agent through killing a broad spectrum of pathogenic microbes (viruses, fungi, and bacteria) [25] and has a suppressive effect on inflammatory cytokines [26]. Khanh Hoang et al. reported new findings regarding the effects of TauCl treatment on pulmonary and systemic inflammation in lipopolysaccharide (LPS)-induced pneumonia in high-fat-diet-induced obese mice [27]. In this model, TauCl treatment attenuated lung edema, accompanied by the inhibition of the gene expression and serum levels of the proinflammatory cytokines IL-6 and TNFα. Furthermore, the skeletal muscle wasting associated with LPS-induced pneumonia was alleviated by the TauCl treatment. 

In conclusion, the newest insights reported in this Special Issue confirm the many various biological roles of taurine. One of the reasons for its various roles could be its simple and specific structure that is very similar to the structures of other β-amino acids with amino and sulfo groups, and it has actually been proved to be an agonist for neuroreceptors of the γ-amino acid GABA and α-amino acid glycine. Moreover, taurine’s properties could be explained considering the amino group itself, which plays a relevant role in the conjugative and/or transfer reactions with metabolites including bile acids, hypochlorous acid, fatty acids, and mitochondrial transfer RNA, and, last but not least, the osmoregulation and detoxification properties, based on the high hydrophilicity of taurine’s sulfo group.
